# Macular hole surgery recovery with and without face-down posturing: a meta-analysis of randomized controlled trials

**DOI:** 10.1186/s12886-019-1272-1

**Published:** 2019-12-21

**Authors:** Ting Ye, Ji-guo Yu, Lin Liao, Lan Liu, Ting Xia, Lei-lei Yang

**Affiliations:** 1 Department of Ophthalmology, Wuhan Fourth Hospital; Puai Hospital, Tongji Medical College, Huazhong University of Science and Technology, Wuhan, 430033 Hubei Province China; 2 Department of Ophthalmology, the Central Hospital of Wuhan, Tongji Medical College, Huazhong University of Science and Technology, Wuhan, 430014 Hubei Province China

**Keywords:** Macular hole, Closure rate, Face down, Posturing, Randomized controlled trials, Meta-analysis

## Abstract

**Background:**

After pars plana vitrectomy with internal limiting membrane (ILM) peeling and gas tamponade, patients are often required to remain in a face-down position (FDP) to allow the gas bubble to push against the macular hole (MH) to promote hole closure. However, this position may be uncomfortable and inconvenient for the elderly and those with medical comorbidities; it may also lead to certain postoperative complications. Hence, this study aimed to evaluate and compare the effect of postoperative FDP and non-face-down position (nFDP) on the closure rate of MHs following MH surgery.

**Methods:**

Randomized controlled trials (RCTs) were selected through an electronic search of the Cochrane Library, Pubmed, and Embase databases. Trial eligibility and risk of bias were assessed according to Cochrane review methods. The primary measures included overall MH closure rate and subgroup analysis based on MH size. Pooled odds ratios (ORs) and 95% confidence intervals (CIs) were estimated. Statistical analysis was performed using RevMan 5.0 software and Stata software 15.0.

**Results:**

Five RCTs composed of a total of 183 eyes in the FDP group and 175 eyes in the nFDP group were included in this meta-analysis. Statistical meta-analysis revealed that the overall MH closure rate in the FDP group was significantly higher than that in the nFDP group (OR = 2.27, 95% CI: 1.02 to 5.05, *P* = 0.04). For MH sizes smaller than 400 μm, the subgroup meta-analysis indicated that the closure rate of the FDP group was not significantly higher than that of the nFDP group (OR = 1.32, 95% CI: 0.39 to 4.49, *P* = 0.66). However, when MH size was larger than 400 μm, there was a significantly higher closure rate in the FDP group (OR = 2.95, 95% CI: 1.10 to 7.94, *P* = 0.03).

**Conclusions:**

Our results provide evidence that a face-down postoperative position seems to be unnecessary when MHs are smaller than 400 μm but may be highly recommended for MHs larger than 400 μm. Further RCTs with large sample sizes are warranted to validate these findings in future.

## Background

Pars plana vitrectomy with internal limiting membrane (ILM) peeling and gas tamponade is an important surgical technique for the closure of a full-thickness macular hole (MH) [1, 2]. After surgery, patients are often required to remain in a face-down position (FDP) to allow the gas bubble to push against the MH to promote hole closure [3, 4]. However, this positioning may be uncomfortable and inconvenient for the elderly and those with medical comorbidities such as arthritis or osteoporosis and can be associated with some postoperative complications such as Ulnar nerve palsies [5] and acute angle closure glaucoma [6]. Meanwhile, it has been reported that prone posturing following MH surgery provides no functional or anatomical benefit [7]. Therefore, studies have been conducted to evaluate whether the non-face-down position (nFDP) is also effective in promoting MH healing [8–10]. A paper by Rubinstein et al. reported that MH surgery without face-down posturing provides anatomical and functional outcomes compared to those with prone posturing [11]. Yagi et al. reported that vitrectomy with ILM peeling and SF6 gas tamponade for MHs without face-down positioning achieved favorable hole closure rates [12]. Mittra et al. reported sustained postoperative face-down positioning may not be necessary because 93% of eyes achieved successful hole closure with prone positioning for only 1 day [13]. Notably however, the FDP has not been completely replaced by the nFDP. Although some vitreoretinal surgeons have reduced the duration of the FDP, they have not entirely abandoned this procedure. Guillaubey et al. maintain that postoperative face-down positioning is highly recommended in holes larger than 400 μm [14]. Whether the FDP or nFDP is recommended following MH surgery and under what circumstances the FDP is required remains unclear and controversial; thus we performed a meta-analysis of randomized controlled trials (RCTs) to evaluate whether the FDP is necessary for recovery from MH surgery.

## Methods

### Search strategy

This meta-analysis was conducted in accordance with the Cochrane Handbook for Systematic Reviews of Interventions and Preferred Items for Systematic Reviews and Meta-Analysis (PRISMA) Statement. A literature search of the PubMed, EMBASE, and Cochrane Library databases up to 10 January 2019 was performed to identify relevant studies. The following terms were used for the searches: “macular hole,” “face down,” “no face down,” “prone positioning,” “supine positioning,” “nonsupine positioning,” “position,” and “posturing.” Results from the electronic databases were imported into a reference management program (EndNote X4; Thomson Reuters, New York, NY, USA). After duplicate articles were deleted, two authors (T.Y. and J.G.Y) read the titles and abstracts of all papers to remove irrelevant reports. Afterwards, the remaining potentially relevant reports were assessed for eligibility by reading the full text based on the inclusion and exclusion criteria described below. In addition, the reference lists from the full-text studies were also searched for additional eligible trials.

### Inclusion and exclusion criteria

The following inclusion criteria were used: (1) RCTs involving MH patients; (2) studies comparing FDPs versus nFDPs (seated or non-supine) after MH surgery; (3) studies where outcome measures included the MH closure rate; and (4) studies in which the surgical technique applied ILM peeling and gas tamponade. The exclusion criteria included studies with insufficient data, not applying ILM peeling, or reporting on silicone oil tamponade, as well as retrospective studies, case reports, and review articles. Two reviewers (L.L.Y. and [L.L]1.) separately evaluated the studies based on the inclusion criteria and discrepancies were resolved through discussion.

### Data extraction

Two reviewers ([L.L]2. and T.X.) independently extracted data from each of the included studies. The extracted data included first author, year of publication, country, study design, number of eyes, mean age, sex, MH size, gas used, ILM peeling, posturing period, and follow-up period. Any discrepancies were resolved by discussion and analysis with another author (L.L.Y.).

### Quality assessment

The Cochrane Risk of Bias assessment tool was applied to assess the risk of bias in evaluating the quality of the RCTs included. Seven domains concerning the quality of the RCTs were observed: 1) random sequence generation, 2) allocation concealment, 3) blinding of participants and personnel, 4) blinding of outcome assessment, 5) incomplete outcome data, 6) selective reporting, and 7) other bias. Each domain was graded into “low risk of bias,” “high risk of bias,” and “unclear risk of bias.” Two reviewers (L.L.Y. and T.Y.) independently evaluated the studies using this tool and disagreements were resolved via discussion.

### Statistical analysis

Statistical analysis was performed using RevMan software (version 5.3; Cochrane Collaboration, Oxford, UK) and Stata software version 15.0 (Stata Corp., College Station, TX). Odds ratios (ORs) were used in the comparisons of dichotomous variables. All statistical analyses were conducted with 95% confidence intervals (CIs). The heterogeneity of studies was assessed using the Chi-square test, with *p* < 0.05 and I^2^ > 50% indicating significant heterogeneity [15]. Heterogeneity was considered to be low when I^2^ ≤ 50%, in which case data were analyzed using the fixed-effects model. Otherwise, the random-effects model was used when I^2^ > 50% [16]. Potential publication bias was examined via visual inspection of a funnel plot [17]. A *P* value < 0.05 was considered to be statistically significant.

## Results

### Search results and characteristics of the studies

**Fig. 1:**
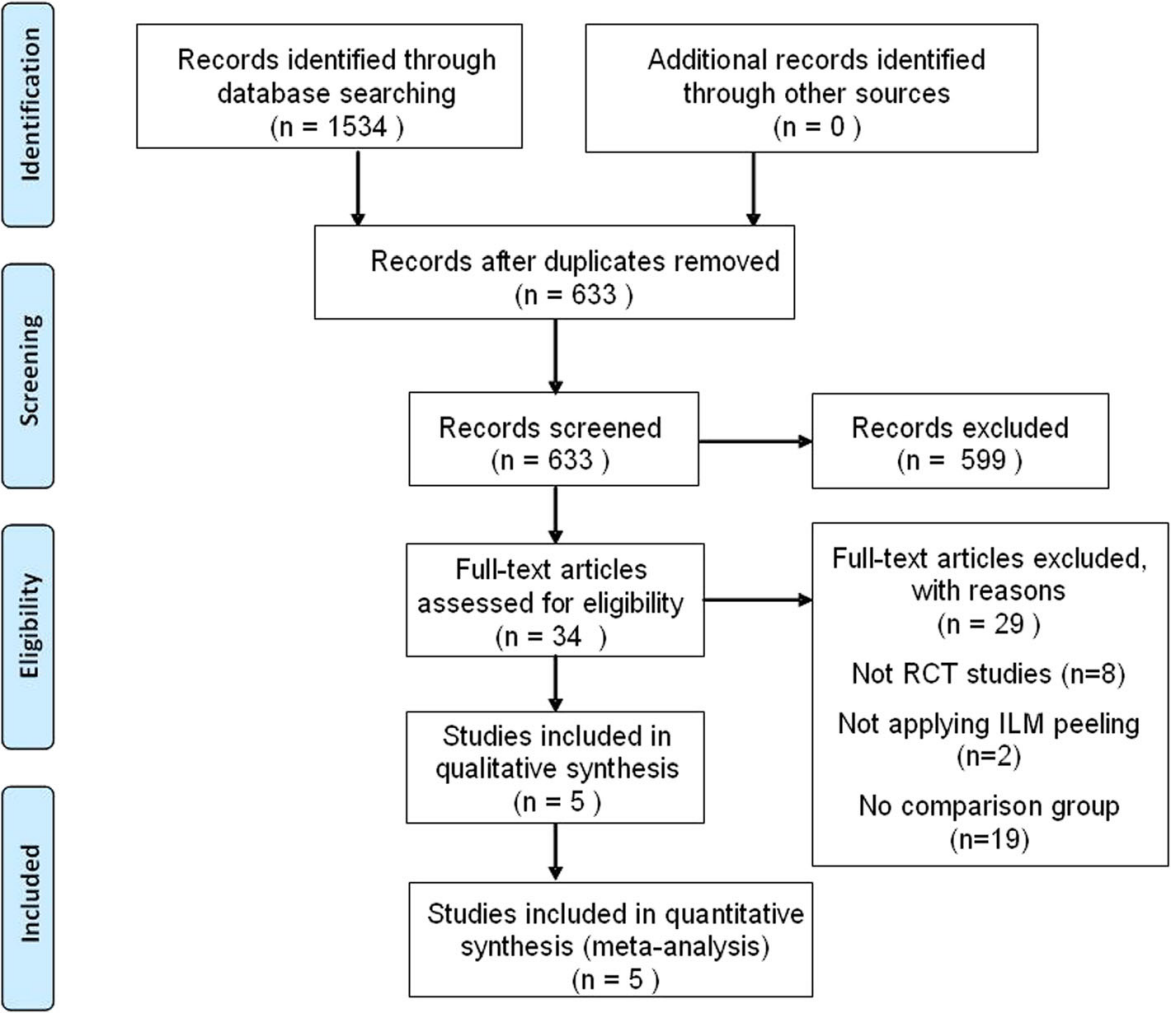
Flow chart of the literature search

**Fig. 2:**
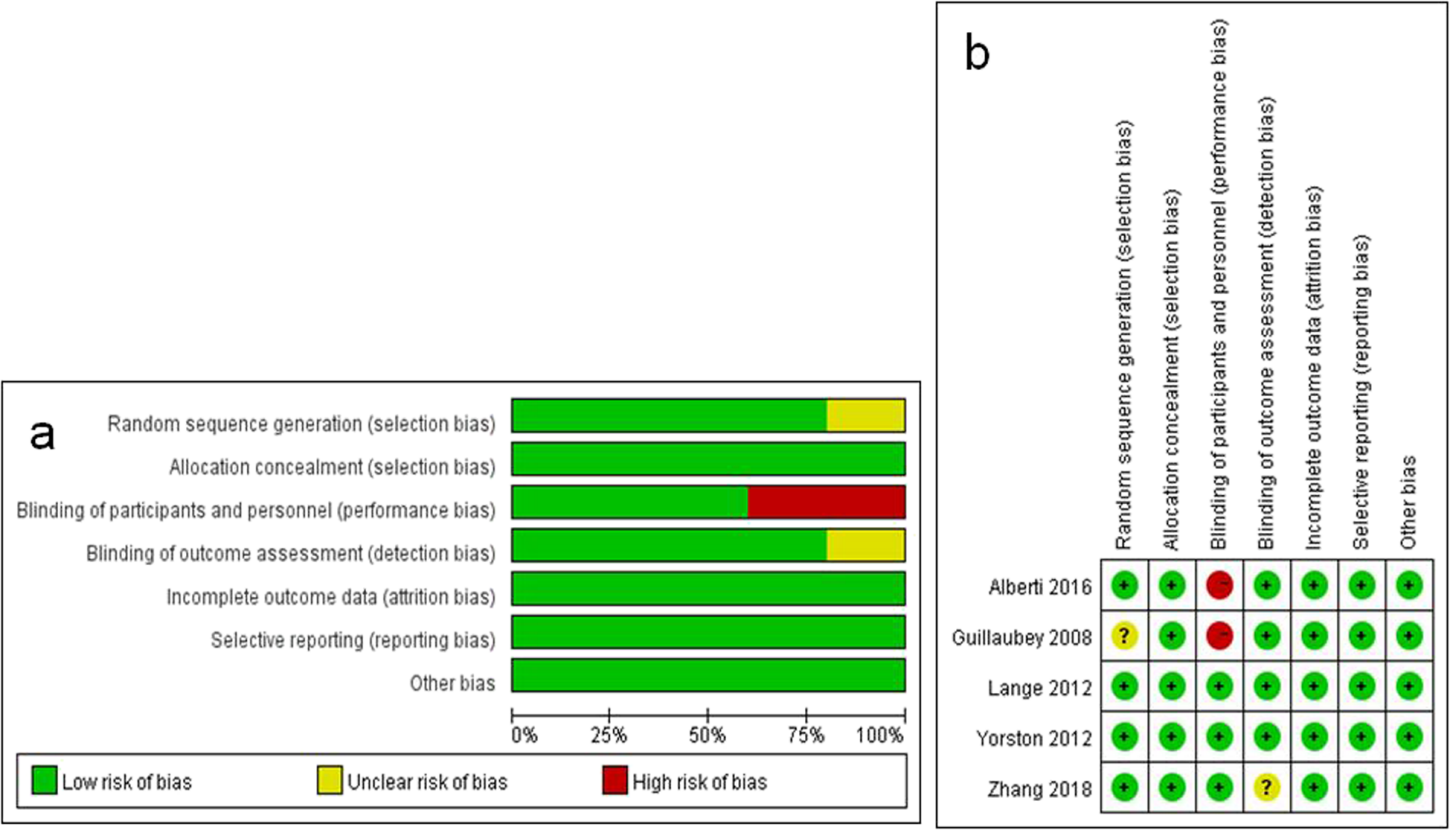
Assessment of the risk of bias in included studies. **a** Risk of bias graph: Review authors’ judgments about each “Risk of bias” item presented as percentages across all included studies; **b** Risk of bias summary: the detailed risk of bias values for each article. +: low risk of bias; −: high risk of bias;?: unclear risk of bias

A total of 1534 records were identified through database searching. There were 633 records left after duplicates were removed, of which 599 reports were excluded after reading the title and abstract. This resulted in a total of 34 reports warranting evaluation for eligibility by reading the full-text. Of these 34 reports, 8 were not RCTs, 2 did not apply ILM peeling, and 19 did not include a comparison group, and were excluded from the meta-analysis as they did not meet the inclusion criteria. The remaining 5 RCTs were included in the final meta-analysis [14, 18–21]. The trial selection process is summarized in Fig. 1. The included studies comprised a total of 358 eyes, of which 183 were in FDP group and 175 in the nFDP group. They were published between 2008 and 2018. The baseline characteristics of each included study are shown in Table 1 and the risk of bias assessment is summarized in Fig. 2. Overall, the included studies were at low risk for bias.

**Table 1:** Summary of the characteristics of the included studies

Author (Year)	Country	Study design	No. of eyes		Mean Age (years)		Gender (Male/Female)		MH size		Gas	ILM peeling	Posturing period	Follow-up period
			FDP	nFDP	FDP	nFDP	FDP	nFDP	< 400 um (F/N)	> 400 um (F/N)				
Guillaubey/2008 [14]	France	RCT	78	72	69.0	68.0	27/51	25/47	37/33	41/39	SF6/C2F6/C3F8	Yes	5 days; 8 h/day	6 months
Lange/2012 [18]	UK	RCT	15	15	66.8	71.0	6/9	4/11	4/5	11/10	C3F8	Yes	10 days; 50 min/hr	6–8 weeks
Yorston/2012 [19]	UK	RCT	16	14	71.1	68.0	1/15	0/14	10/9	6/5	C3F8	Yes	10 days; 50 min/hr	6 months
Alberti/2016 [20]	Denmark	RCT	34	34	69.8	69.3	14/20	13/21	18/13	16/21	C3F8	Yes	3 days; 10 h/day	3 months
Zhang/2018 [21]	China	RCT	40	40	62.35	62.85	7/33	7/33	33/33	7/7	C3F8	Yes	3 days; 16 h/day	3 months

*UK* United Kingdom, *RCT* randomized controlled trial, *FDP* face-down position, *nFDP* non-face-down position, *F* FDP, *N* nFDP, *hr.* hour

### Outcomes of meta-analysis

#### Overall MH closure rate

**Fig. 3:**
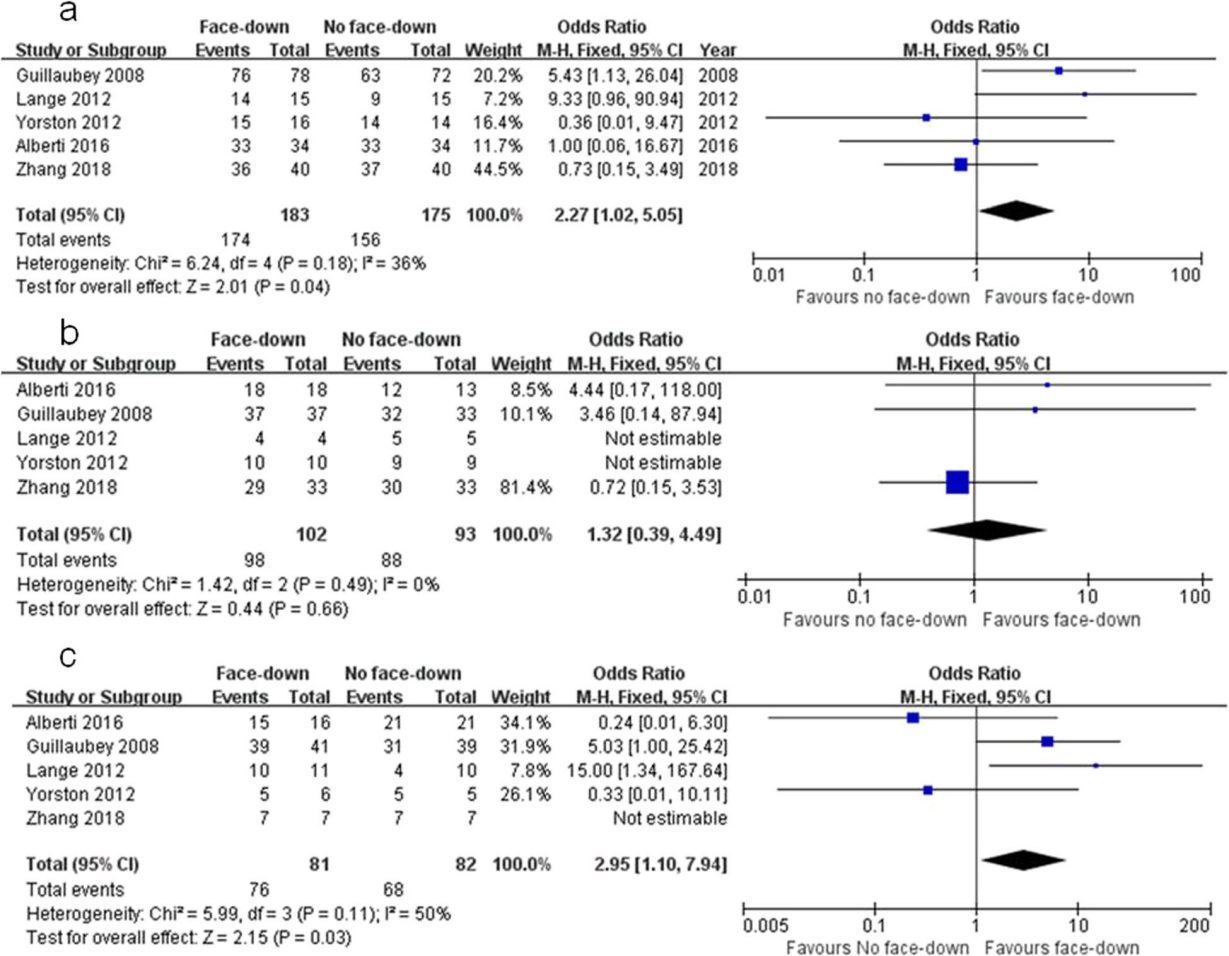
Forest plots of MH closure compared between the FDP and nFDP groups in overall and subgroup comparisons. **a**: overall macular hole closure; **b**: macular hole smaller than 400 μm; **c**: macular hole larger than 400 μm)

Overall MH closure rate was compared between the FDP and nFDP groups across five studies. No statistical heterogeneity was found (I^2^ = 36%). Therefore, the data were analyzed using a fixed-effects model. Meta-analysis of these data showed that the overall MH closure rate in the FDP group was significantly higher than that in the nFDP group (OR = 2.27, 95% CI: 1.02 to 5.05, *P* = 0.04; Fig. 3a).

#### MH size smaller than 400 μm

Subgroup meta-analysis of MH closure rate was performed based on MH size. Five studies, in which the MH size was smaller than 400 μm, were pooled together, including 102 eyes in the FDP group and 93 eyes in the nFDP group. No significant heterogeneity (I^2^ = 0%) was found across the studies, so the data were pooled through the fixed-effects model. The meta-analysis of these data showed that there was no statistically significant difference in MH closure rate between the FDP and nFDP groups (OR = 1.32, 95% CI: 0.39 to 4.49, *P* = 0.66; Fig. 3b).

#### MH size larger than 400 μm

Five studies, in which the MH size was larger than 400 μm, were pooled together, including 81 eyes in the FDP group and 82 eyes in the nFDP group. No significant heterogeneity (I^2^ = 50%) was found across the studies, so the data were pooled through the fixed-effects model. The meta-analysis of these data showed a significantly higher MH closure rate in the FDP group than in the nFDP group (OR = 2.95, 95% CI: 1.10 to 7.94, *P* = 0.03; Fig. 3c).

#### Publication bias

**Fig. 4:**
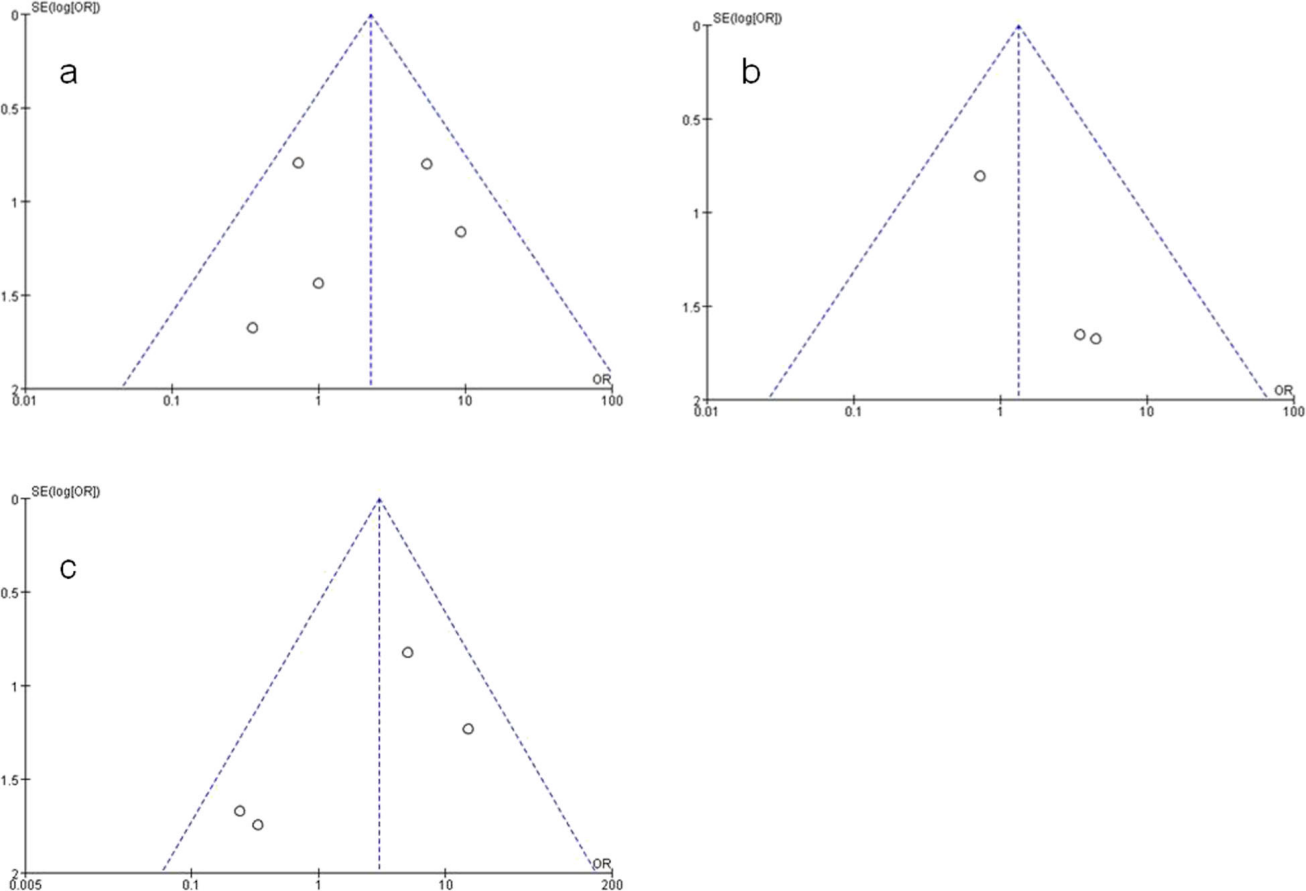
A funnel plot for evaluating publication bias. **a**: overall macular hole closure; **b**: macular hole smaller than 400 μm; **c**: macular hole larger than 400 μm)

Potential publication bias was assessed by funnel plot and Egger’s test. The funnel plots indicated that there were no obvious asymmetries in any of the studies included (Fig. 4). Moreover, Egger’s test showed that there were no obvious publication biases in the analysis of the overall MH closure rate (*P* = 0.704) and for MH sizes larger than 400 μm (*P* = 0.290); however, a publication bias was observed for MH sizes smaller than 400 μm (*P* = 0.046).

## Discussion

The current meta-analysis of five RCTs showed that the overall MH closure rate in the FDP group was significantly higher than that in the nFDP group. The subgroup meta-analysis also demonstrated significant differences in the MH closure rate between the two groups with MH sizes larger than 400 μm but not with MH sizes smaller than 400 μm. Our meta-analysis strongly suggests that the success rate of MH healing is related to the face-down position when MH size is larger than 400 μm. Therefore, it is necessary to keep these patients face down after MH surgery with gas tamponade. However, for patients with MH sizes smaller than 400 μm, postoperative face-down posture is not mandatory, as long as supine positioning is avoided. This finding will help individuals with small MHs (≤ 400 μm) who are hesitant of the surgery because of the postoperative prone position constraint seek surgical repair for MH. Furthermore, the adoption of a non-face-down position may spare patients painful discomfort and avoid certain complications, and in certain cases, shorten the duration of hospitalization or absence from work [22]. For those scholars who think postoperative non-supine position adoption is adequate for all MH patients after surgery [9, 10, 23], we suggest that the position required according to the MH size is more appropriate rather than blindly abandoning the postoperative position.

The formation of MHs is caused by the anteroposterior and tangential traction from posterior vitreous detachment and the proliferation of glial cells [24, 25]. The traction can be removed by pars plana vitrectomy with ILM peeling [26]. In the case of gas filling, a MH smaller than 400 μm was enough to achieve successful anatomic healing without the need for the prone position [27]. This finding may depend on the hydration theory that the subfoveal subretinal fluid is pumped out by the retinal pigment epithelium quickly, once traction is relieved [28]. However, for MHs larger than 400 μm, although the tangential force was removed by ILM peeling, the MH was also difficult to heal. Because the distance between the broken ends of the retina is so large, it requires a strict prone position to ensure that the gas bubble, under a sufficient or partial absorption state, keeps the intraocular gas-macula contact extended, drawing the edges of the hole into apposition with each other, thus providing a scaffold for the migration of glial cells, blocking fluid entry into the hole and moving the subretinal fluid to reattach the retina. These processes contribute to successful anatomic healing [8, 21, 29]. This may explain why, for patients with large MHs, the successful healing rate of the FDP group was higher than that of the nFDP group. No significant difference was found in the MH closure rate of patients with small MHs between the FDP and nFDP group.

Furthermore, the gas will be gradually absorbed with time, and the buoyancy will be weakened after partial gas absorption. Within 3 days, 30% of the gas will be absorbed [21], which does not affect the healing of MHs smaller than 400 μm. This may be due to the removal of the traction force by ILM peeling which is important for hole healing in addition to the top pressure effect of the gas. However, for the larger holes, the traction force removal is not enough to make the hole close gradually. Gas-macula contact and upward mechanical force on the macula are required for effective tamponade. The retina must be acted on by an external force for it to be displaced, thus apposing the MH edges [9]. Partial gas absorption with time will affect the buoyancy, and therefore the face-down position is needed to keep the remaining gas buoyant against the MH and to sustain apposition of the gas bubble to the inner surface of the MH to facilitate closure. In other words, the upward mechanical force of the gas on the macula, blocking the action of fluid entry into the hole and providing a scaffold/bridge for gliosis play an important role in the healing process of large holes.

Previous studies have reported that the healing of MHs begins within 24 h after the surgery, and the bridge configuration occurs around 3 days thereafter [30, 31]. The MHs were basically healed within 3 days after surgery, and those that were not healed within 3 days were still open during the 3-month follow-up [3, 21]. All of the patients from the studies included in this meta-analysis were in the prone position for 3 days or more, so the comparison of the closure rate of the MHs with sizes larger than 400 μm in the FDP and nFDP groups after MH surgery is credible. In short, patients with MHs larger than 400 μm should keep the face-down positioning strictly for 3 days after MH surgery. For patients who MH closure is not achieved within 3 days in an FDP, continuing an FDP will not increase healing. A strict prone position for 3 days after the operation is not a long time and should have little impact on systemic complications, which is conducive to improving the compliance of patients.

The studies included in this meta-analysis did not provide the same FDP time following surgery. It should be noted that because many MH patients are elderly, compliance to the prone position is usually not high due to systemic factors, and therefore, the five included studies only required patients to keep the FDP for a few hours per day. In addition, although patients are advised to remain face down everyday by their surgeon, they cannot be supervised after they leave the clinical setting. Hence, there may be some patients showing low compliance who did not spend sufficient time in the FDP to meet treatment requirements, which may have affected the accuracy of the study results. As there is currently no consensus on the number of hours necessary for patients with larger MHs to remain in the FDP daily, RCT studies with large sample sizes investigating this topic are necessary in future. In addition, although MH duration prior to surgical intervention is an important variable affecting closure rate, there was no significant difference in MH duration between the FDP and nFDP groups in the included studies; hence, we believe that MH duration did not influence the results of our meta-analysis.

There were some limitations to this study. First, all of the included studies had small sample sizes, and there is a lack of data with large sample sizes from multi-center RCTs in our research. Second, this meta-analysis was restricted to studies published in indexed journals and we did not search for unpublished studies, original data, or papers published in non-English languages, which may have led to publication bias. Meanwhile, two studies [18, 19] reported 100% MH closure rate in the FDP and nFDP groups for MH sizes smaller than 400 μm, which resulted in a reduction in the number of publications included in the bias analysis. This methodology resulted in an overemphasis on positive results and neglect of negative results, potentially accounting for the publication bias described. Third, although Zhang et al. used the inverted ILM flap technique when performing surgery on MHs larger than 400 μm, the rate of use of the inverted ILM flap technique was not significantly different between the FDP and nFDP groups. However, 100% MH closure was achieved in patients undergoing the inverted ILM flap technique with and without face-down posturing. Therefore, although we believe that the use of different surgical techniques did not affect the results of our meta-analysis, it remains a possible source of bias. Fourth, we did not perform subgroup meta-analysis for visual acuity between the two groups due to the different definitions of visual acuity improvement among the five included studies. The images of the repaired MHs were categorized into three patterns (U-type, V-type, and W-type) by optical coherence tomography [32]. We were also not able to perform a subgroup analysis of the closure type due to lack of relevant data. Lastly, the area of ILM peeling, air or longer-acting gas tamponade and whether the lens were removed may also have influenced the closure rates [33]. However, we were unable to find sufficient data to investigate these parameters. Therefore, longitudinal in-depth RCTs with large sample sizes evaluating the aforementioned parameters are required in future to investigate differences between the two groups.

## Conclusion

In conclusion, this meta-analysis provides sufficient evidence that a non-face-down postoperative position is not inferior to a face-down position when the MH is smaller than 400 μm. Our findings also confirm that a face-down postoperative position is highly recommended in MHs larger than 400 μm. The results of this study may help elderly patients to alleviate posturing difficulties and avoid systemic complications related to FDPs when their MH is smaller than 400 μm, as we found it did not affect the closure rate. Further RCTs with large sample sizes are warranted to validate these findings in future.

## Data Availability

The datasets analyzed during the current study are available from the corresponding author on reasonable request.

## Abbreviations

CIs: Confidence intervals
F: FDP
FDP: Face-down position
hr.: Hour
ILM: Internal limiting membrane
MH: Macular hole
N: NFDP
nFDP: Non-face-down position
ORs: Odds ratios
RCTs: Randomized controlled trials
UK: United Kingdom
